# Minority Carrier Lifetime Measurements for Contactless Oxidation Process Characterization and Furnace Profiling

**DOI:** 10.3390/ma12010190

**Published:** 2019-01-08

**Authors:** Christian Bscheid, Christian R. Engst, Ignaz Eisele, Christoph Kutter

**Affiliations:** 1 Fraunhofer Research Institution for Microsystems and Solid State Technologies (EMFT), Hansastrasse 27d, 80686 Munich, Germany; Christian.Bscheid@emft.fraunhofer.de (C.B.); Ignaz.Eisele@emft.fraunhofer.de (I.E.); Christoph.Kutter@emft.fraunhofer.de (C.K.); 2 Institute of Physics, Universität der Bundeswehr München, Werner-Heisenberg-Weg 39, 85577 Neubiberg, Germany

**Keywords:** lifetime, high-resistivity, float zone, furnace profiling, oxide characterization

## Abstract

Contactless minority carrier lifetime (lifetime) measurements by means of microwave detected photoconductivity are employed for oxidation process characterization and furnace profiling. Characterization is performed on oxidized float zone substrates with high resistivity and outstanding bulk quality, suggesting that the measured effective lifetime is strongly dominated by interface recombination and therefore reflects the oxide quality. The applied approach requires neither test structures nor time consuming measurements and is therefore of particular interest if high throughput is required. The method is used to investigate the impact of oxidation furnace leakage as well as to analyze the oxidation homogeneity across a horizontal oxidation furnace. For comparison, capacitance-voltage measurements are conducted to characterize the oxide properties. It is found that any type of furnace leakage, which induces fixed oxide charges as well as interface states, has a heavy impact on the measured effective lifetime, especially on the shape of generation rate dependent lifetime curves. Furthermore, a distinct lifetime decrease towards the tube door of the oxidation furnace could be observed. The latter is even detectable in an ideal oxidation process, generating high quality oxides. Besides plain equipment characterization, the presented approach is suitable to optimize the oxidation process itself regarding different parameters like temperature, gas flow, pressure, or process time.

## 1. Introduction

Thermally grown silicon dioxide (SiO_2_) plays an essential role in today’s semiconductor device technology. Easy fabrication, high dielectric strength, and an outstanding degree of chemical passivation makes SiO_2_ the dielectric of choice for many silicon (Si) based devices. However, the actual oxide quality, which is achieved across the oxidation furnace, strongly depends on the correct choice of process parameters as well as on the equipment status. To monitor the oxidation process regarding important electrical oxide properties, a variety of characterization techniques is available.

Probably the most common approach to gain information about the electrical oxide quality are capacitance-voltage measurements, which are performed on metal oxide semiconductor (MOS) capacitors [1]. In this method, a voltage is applied to the MOS structure in order to drive the near-surface condition from inversion to accumulation or vice versa. The resulting capacitance-voltage curves can be used to extract a variety of parameters, whereby the fixed oxide charge *Q*_ox_ as well as the Si/SiO_2_ interface state density *D*_it_ are typically of particular interest. However, both capacitance-voltage measurements and the fabrication of MOS capacitors are very time-consuming, which is a severe drawback, especially if high throughput is required. In addition, the fabrication of MOS structures often requires high temperature process steps like post-implantation or forming gas annealing, which may alter the oxide properties drastically, i.e., information about the plain oxidation process is not easy to obtain.

A different technique to characterize the oxide quality are minority carrier lifetime (lifetime) measurements, which are performed in dependence of the near-surface condition. While sweeping the latter from accumulation to inversion, a distinct lifetime dip can be observed, which is directly correlated to *Q*_ox_ and *D*_it_ [2]. Measurements may be performed by means of test structures like gate-controlled point-junction diodes [2] and metal insulator systems with transparent gate [3] or completely contactless using the corona-charged surface (CCS) approach [4]. While the first two techniques suffer from the same disadvantages as capacitance-voltage measurements, i.e., the fabrication of test structures, the CCS approach is predestined to characterize the plain oxidation process. However, corona charging is still time-consuming and therefore unsuited if high throughput is required. Especially if only qualitative information, i.e., the process uniformity across the furnace, rather than quantitative values like *Q*_ox_ and *D*_it_, are of interest.

Lifetime measurements may also be performed on oxidized substrates without any external modulation of the near-surface condition. From such measurements typically effective lifetimes are obtained, which depend on both bulk and interface recombination [5]. However, with the correct substrate choice, the measured effective lifetime is heavily dominated by interface recombination and therefore reflects the oxide quality [6]. In the present work, this approach is applied to investigate the oxidation homogeneity across single wafers as well as the entire oxidation furnace. Lifetime measurements are performed on oxidized float zone (FZ) substrates with high resistivity, which are known for extremely high bulk lifetimes. Due to this fact, the measured effective lifetime is clearly dominated by surface recombination, even if thermally grown oxides with very low interface densities are used for surface passivation [6]. The applied approach does not require the fabrication of test structures and is therefore ideally suited to characterize every single wafer of a batch process, especially if only qualitative information about the process homogeneity is of interest. In addition, the quality of the plain oxidation process is obtained since no additional high temperature process steps are performed between oxidation and the actual measurements.

## 2. Materials and Methods

In order to prove that the lifetime measurements refer to the characteristics of the oxidation furnace and not to different substrate properties, a set of high-resistivity FZ substrates served as starting material for the present work. The wafers have similar resistivity ranges as well as identical surface orientation, but were produced out of three different ingots. Such material is typically used for detector or power applications and therefore expected to have an outstanding crystal quality. In addition, slightly doped substrates are advantageous for contactless lifetime measurement, since extremely high signal to noise ratios are obtained. Detailed material information can be found in Table 1.

A thermal dry oxide of approximately 200 nm was grown on every wafer using a horizontal oxidation furnace. After the oxidation, in situ post oxidation annealing under nitrogen atmosphere was performed for 15 min in order to reduce fixed oxide charges as well as Si/SiO_2_ interface states. Both oxidation and post oxidation annealing were carried out at T = 1030 °C at atmospheric pressure. Ramp up and cool down were performed under oxygen and nitrogen atmosphere, respectively. Before every oxidation, a pre-oxidation clean was conducted in the furnace. All wafers were oxidized as delivered by the manufacturer, i.e., neither surface preparation nor cleaning was performed.

The oxidation process was split into five different runs, whereby every run contained only wafers from one single batch. In order to investigate the impact of furnace leakages on the oxide properties, two runs (*α* and *β*) were performed using a defective tube door sealing, i.e., leakage at the tube door was intentionally provoked. The other oxidation runs (*γ*, *δ* and *ε*) were conducted using a proper tube door sealing. Apart from the intentionally provoked leakage, all process parameters were kept constant for every oxidation run. A detailed description and loading plan of each oxidation can be found in Table 2. To avoid any systematic effect, which may be caused by material deviations across the ingot, the wafers were placed randomly with respect to the wafer number, i.e., ingot position, in the quartz boat. The latter has 50 slots, whereby slot 1 is the innermost quartz boat position and slot 50 is located next to the tube door. During the oxidation all slots were occupied, either by dummy wafers or FZ substrates.

**Figure 1:**
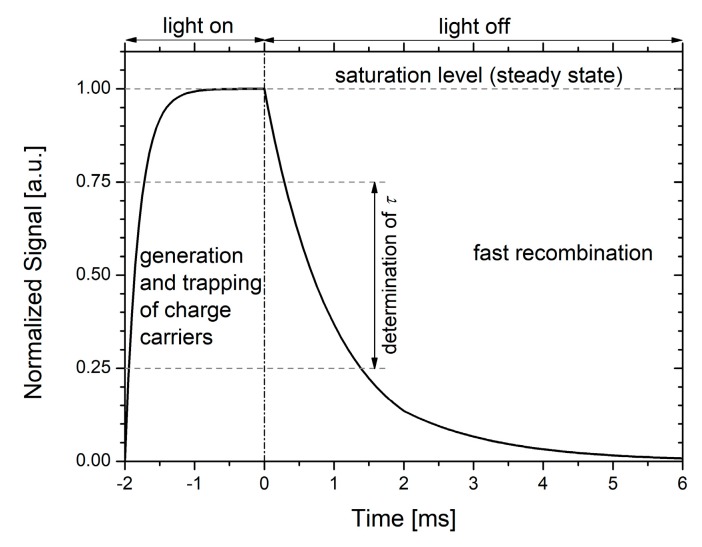
Exemplary microwave detected photoconductivity (MDP) measurement transient.

**Figure 2:**
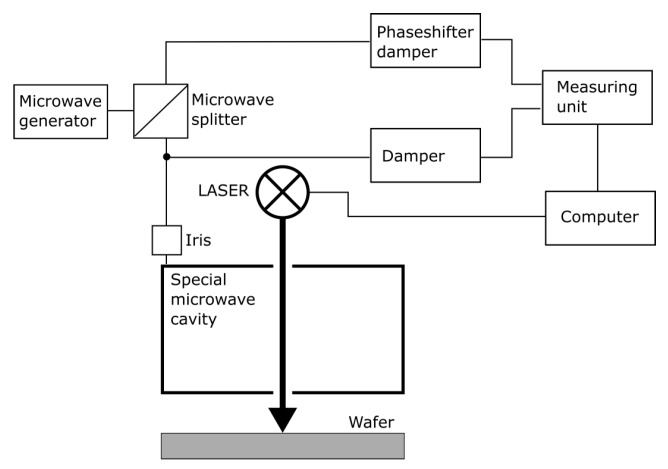
Schematic MDP measurement setup.

Following the oxidation, a detailed lifetime analysis by means of the microwave detected photoconductivity (MDP) technique [7] was conducted on every FZ wafer. In MDP, optical generation of excess carriers is performed until a steady state photoconductivity is reached (see Figure 1). After the optical excitation is switched off, excess carriers decay, and cause a decrease of the measured photoconductivity. The lifetime is calculated from the observed exponential decay, whereby linear regression is performed between 75% and 25% of the signal height (see Figure 1) [8]. Rise and decay of the photoconductivity are measured by microwave absorption, therefore, the wafer to be measured is part of a resonant microwave cavity. Excitation is typically performed by means of different laser diodes. The schematic arrangement of an MDP measurement setup is depicted in Figure 2. A detailed description of the MDP measurement principle can be found in Reference [9].

The measured lifetime is typically an effective lifetime *τ*_eff_, which can be expressed by

(1) $$\frac{1}{\tau_{\mathrm{eff}}}=\frac{1}{\tau_{b}}+\frac{1}{\tau_{s}}$$

where *τ*_b_ is the bulk and *τ*_s_ the surface or interface lifetime [5]. In oxidized FZ material with outstanding crystal quality, the bulk lifetime *τ*_b_ is expected to be significantly higher than the interface lifetime *τ*_s_ [6]. Thus, the measured effective lifetime *τ*_eff_ is limited by surface recombination, which depends on the Si/SiO_2_ interface state density *D*_it_, the fixed oxide charge *Q*_ox_, the capture cross section for electrons *σ*_n_, and holes *σ*_p_ as well as the charge carrier concentration of electrons *n*_s_ and holes *p*_s_ at the surface [2].

The diffusion length L is closely related to the effective lifetime and may be calculated according to the equation [10]

(2) $$L=\sqrt{D\;\tau_{\mathrm{eff}}}$$

Depending on the injection level, *D* is either the minority carrier or the ambipolar diffusion coefficient [5].

All lifetime measurements were performed on a commercially available MDPmap setup (Freiberg Instruments), which enables fast lifetime mappings on wafer scale. Optical excitation was performed by means of IR laser diodes (977 nm) with 0.5 mm spot diameter, which cover an optical generation rate ranging from 1.2 × 10^18^ cm^−3^ s^−1^ to 5.6 × 10^21^ cm^−3^ s^−1^. The optical generation rate *G*_opt_ is calculated according to

(3) $$G_{\mathrm{opt}}=\frac{1}{d}\int_{0}^{d}\alpha \;\Phi \left(x\right)\;dx$$

where *d* is the wafer thickness, *α* the absorption coefficient and Φ(*x*) the optical flux [11].

The effective lifetime across every wafer was mapped with 2 mm raster resolution, whereby an edge exclusion of 5 mm was used. Depending on the wafer diameter, this results in approximately 1590 and 3850 measuring points, respectively. To ensure comparability, all mappings were performed with an optical generation rate of 5.6 × 10^21^ cm^−3^ s^−1^. After the wafer mapping, the median value as well as the 25–75% quantile, also known as interquartile range, were stored and used for further evaluation. In this context, the 25–75% quantile is defined as the difference between the third quartile and the first quartile, i.e., *Q*_0.75_–Q_0.25_.

Additionally, generation rate dependent lifetime measurements were performed at the center of each wafer, i.e., transients are recorded for different optical generation rates and the effective lifetime is subsequently extracted from each transient as shown in Figure 1.

A typical measurement sequence, i.e., wafer mapping as well as generation rate dependent lifetime analysis, takes approximately 5 min.

For capacitance-voltage measurements, circular MOS capacitors with 1 mm^2^ Aluminum/Silicon (Al/Si) gate area were fabricated on several oxidized FZ wafers. Fabrication of MOS structures required the implantation of phosphorus in order to create ohmic contacts to the substrate as well as annealing in nitrogen and forming gas atmosphere. Capacitance-voltage analysis was performed across all test wafers using a B1500A Semiconductor Device Analyzer (Agilent). Measurements were carried out for gate voltages between −4 V and 4 V, either as plain quasi static measurement or by superimposing an alternating current (AC) signal with 5 kHz frequency and 75 mV effective amplitude to the direct current (DC) voltage. Before data evaluation, all capacitance-voltage data were corrected for parasitic effects such as series resistance and offset cable capacitance.

Depending on the applied voltage, different capacitance-voltage characteristics may be obtained. If a plain DC voltage is applied to the MOS capacitor, a so-called quasi static or low frequency behavior is observed. Si/SiO_2_ interface states contribute to the measured low frequency capacitance *C*_lf_ and identical capacitances are observed under inversion and accumulation conditions, which are equal to the oxide capacitance *C*_ox_. If a small high frequency signal is superimposed to the DC bias, the inversion capacitance may be determined by a modulated space charge region and interface traps may not contribute to the measured high frequency capacitance. However, the actual frequency at which high frequency behavior is observed strongly depends on the material properties. High frequency capacitance-voltage characteristics can be used to estimate the semiconductor doping density as well as the flatband voltage V_fb_. The latter is related to the fixed oxide charge *Q*_ox_ by the equation

(4) $$Q_{\mathrm{ox}}=\left(\varphi_{\mathrm{ms}}-V_{\mathrm{fb}}\right)C_{\mathrm{ox}}$$

where *φ*_ms_ is the metal-semiconductor work function difference [5].

The Si/SiO_2_ interface state density *D*_it_ may be calculated from the expression

(5) $$D_{\mathrm{it}}=\frac{1}{q^{2}}\left(\frac{C_{\mathrm{lf}}\;C_{\mathrm{ox}}}{C_{\mathrm{ox}}-C_{\mathrm{lf}}}-C_{s}\right)$$

where *q* is the elementary charge and *C*_S_ is the semiconductor capacitance that is not known a priori. The determination of *C*_S_ requires the knowledge of the relation between the gate voltage *V*_G_ and the surface potential *Φ*_s_, which is obtained by integration of the low frequency capacitance-voltage curve:

(6) $$\Phi_{s}=\int_{V_{G1}}^{V_{G2}}\left(1-\frac{C_{\mathrm{lf}}}{C_{\mathrm{ox}}}\right)dV_{G}+\Delta$$

If integration is started at the flatband condition, i.e., *V*_G1_ = *V*_fb_, the integration constant Δ becomes 0 [5].

## 3. Results

### 3.1. Oxidation Process Characterization

**Figure 3:**
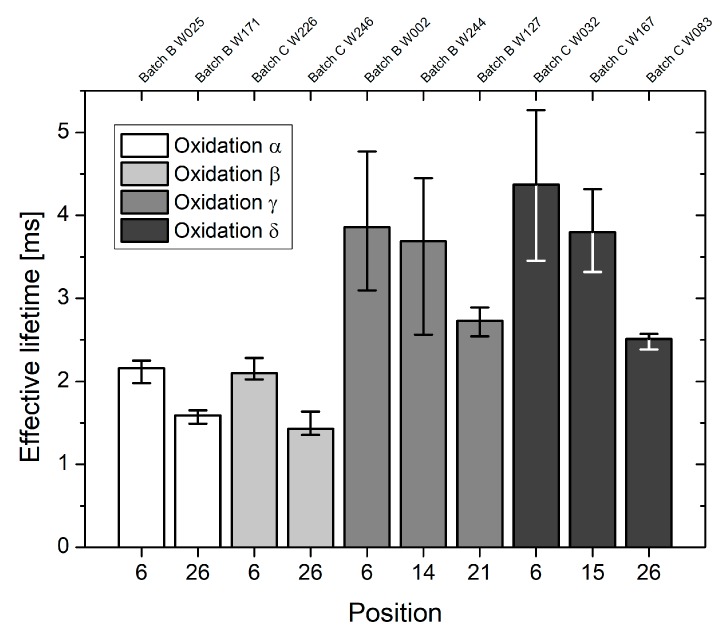
For oxidation runs *α*, *β*, *γ* and *δ*, respectively, the effective lifetime (median value, 25–75% quantile) is shown as a function of the quartz boat position and wafer number. While oxidation runs *α* and *γ* only contained 150 mm wafers from batch B, oxidation runs *β* and *δ* only contained 100 mm wafers from batch C. Quartz boat positions 21 and 26, respectively, are located in direction of the tube door.

The impact of furnace leakage on the effective lifetime across a single wafer is illustrated in Figure 3. Oxidized FZ substrates from the runs *α* and *β*, i.e., with a defective tube door, show a significantly reduced effective lifetime compared to those wafers, which were oxidized during the runs *γ* and *δ*. From Figure 3 it is clearly visible that for every oxidation run a lifetime decrease appears to occur in direction to the tube door. The latter effect will be studied in detail in Section 3.2.

The influence of leakage on the measured effective lifetime is further investigated by means of injection dependent lifetime analysis. Due to observed high lifetimes (see Figure 3), the diffusion lengths, estimated according to Equation (2), strongly exceed the laser spot diameter and it is not possible to calculate the actual injection level [8]. Therefore, throughout this work the effective lifetime is plotted as a function of the optical generation rate instead of the injection level.

**Figure 4:**
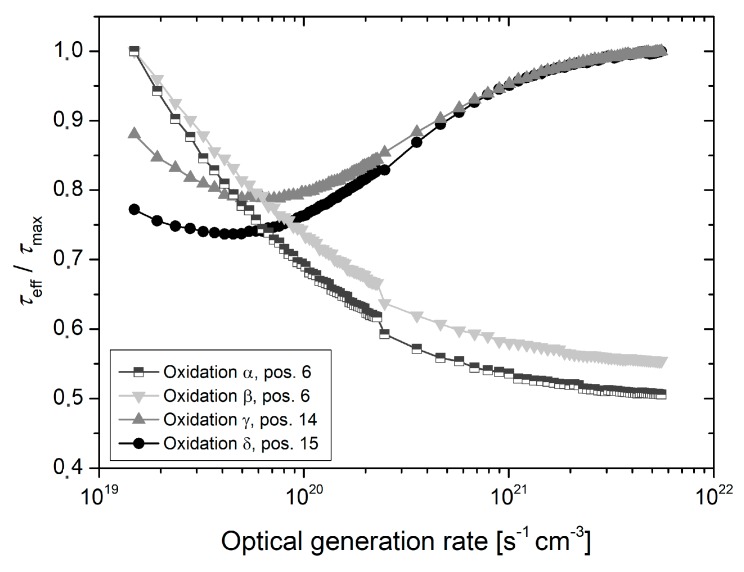
Effective lifetime as a function of the optical generation rate measured in the center of different wafers from the oxidation runs *α*, *β*, *γ* and *δ*, respectively.

Exemplary generation rate dependent lifetime curves, which were recorded in the center of each wafer, are depicted in Figure 4. In the case of the nearly ideal oxidation runs *γ* and *δ*, the highest effective lifetimes are observed for the maximum optical generation rate of 5.6 × 10^21^ cm^−3^ s^−1^. The normalized lifetime is initially decreasing, passes a minimum, and finally increases with declining optical generation rates. For runs *α* and *β* with a leaky tube door, an exact opposite behavior is observed. Throughout the generation rate range, the normalized lifetime increases with declining optical generation rate and the highest lifetimes are observed for the minimum optical generation rate of 1.2 × 10^19^ cm^−3^ s^−1^. It is to mention that the strong impact of furnace leakage on the measured lifetime occurs independent of batch and wafer diameter, i.e., this effect can clearly be attributed to interface recombination.

**Figure 5:**
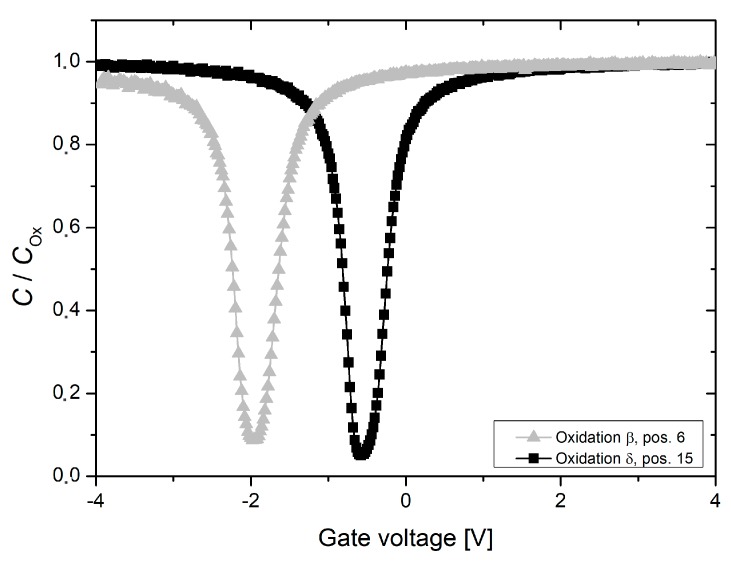
Exemplary results of quasi static capacitance-voltage measurements, which were performed on one test wafer from the oxidation runs *β* and *δ*, respectively. Measurements were carried out at metal oxide semi-conductor (MOS) capacitors (area 1 mm^2^) in the center of each wafer.

Capacitance-voltage measurements, which were performed on MOS structure across one wafer from oxidation run *β*, revealed an average fixed oxide charge density of 1.3 × 10^11^ cm^−2^ in addition to an average Si/SiO_2_ interface state density of about 2 × 10^10^ cm^−2^ eV^−1^ at midgap. It is to mention that annealing in nitrogen and forming gas atmosphere was performed during the fabrication of MOS structures, which may have reduced the fixed oxide charge and Si/SiO_2_ interface state density, respectively [1]. As clearly visible on the exemplary capacitance-voltage curves from Figure 5, the fixed oxide charge as well as the Si/SiO_2_ interface state density at midgap are reduced for oxidation run *δ*. Both values are below the detection limit of the applied characterization methods, suggesting that *Q*_ox_ < 1 × 10^10^ cm^−2^ and *D*_it_ < 1.0 × 10^10^ cm^−2^ eV^−1^ [5].

### 3.2. Furnace Profiling

**Figure 6:**
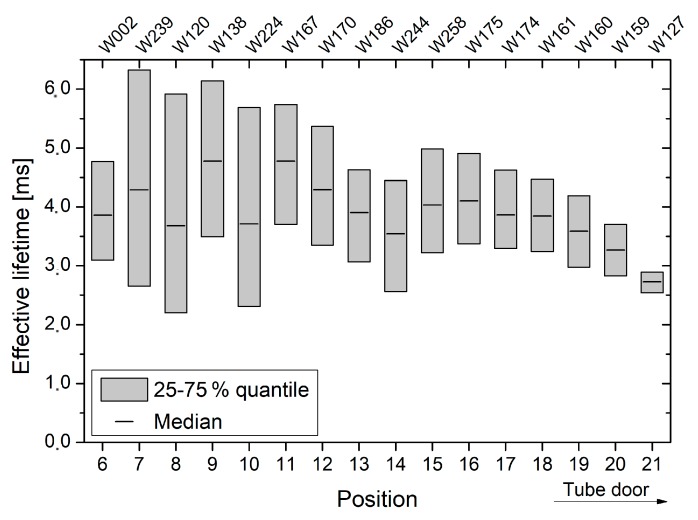
For oxidation run *γ*, effective lifetimes are shown as a function of the quartz boat position and wafer number, respectively. The quartz boat positions 6–21 contained only 150 mm wafers from batch B, whereby quartz boat position 21 is located in direction of the tube door.

Detailed results of oxidation *γ*, which was performed on 150 mm substrates from batch B, are shown in Figure 6. In general, an inhomogeneous lifetime profile is clearly visible. Next to the lifetime decrease towards the tube door, an oscillating behavior is noticeable between quartz boat positions 6–14.

**Figure 7:**
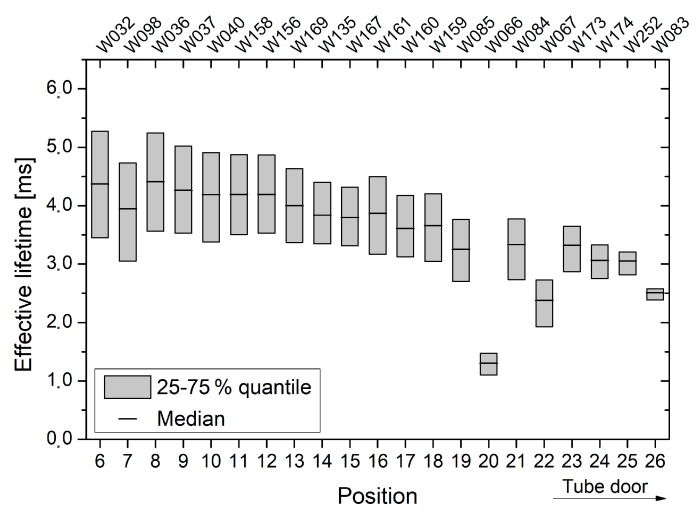
For oxidation run *δ*, effective lifetimes are shown as a function of the quartz boat position and wafer number, respectively. The quartz boat positions 6–26 contained only 100 mm wafers from batch C, whereby quartz boat position 26 is located in direction of the tube door.

A similar picture was observed for oxidation *δ* (see Figure 7). The latter contained 100 mm wafers from batch C, which were intentionally placed up to quartz boat position 26. Starting form quartz boat position 8, the measured effective lifetime is continuously decreasing with the quartz boat positions, whereby slight lifetime oscillations are visible. Except for the wafers, which were placed in quartz boat positions 20 and 22, the observed lifetimes are comparable to those obtained from oxidation *γ*.

**Figure 8:**
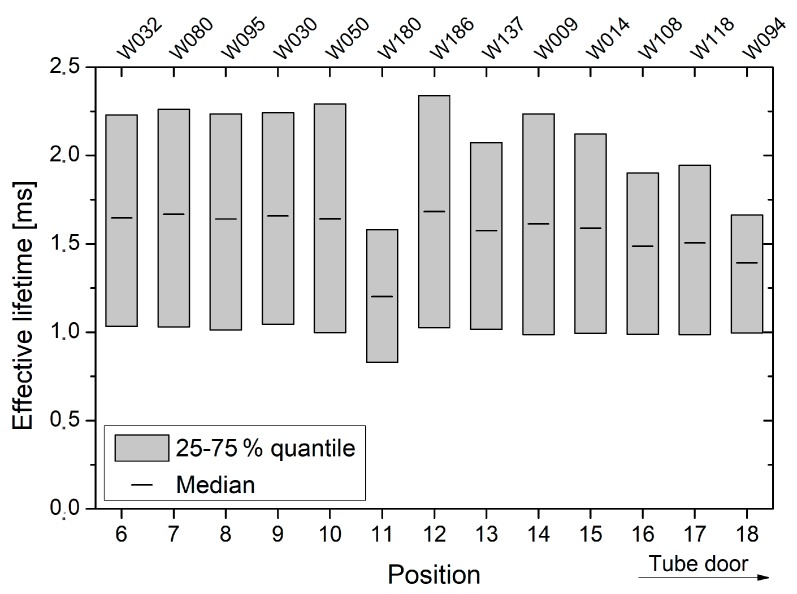
For oxidation run *ε*, effective lifetimes are shown as a function of quartz boat position and wafer number, respectively. The quartz boat positions 6–18 contained only 150 mm wafers from batch A, whereby quartz boat position 18 is located in direction of the tube door.

For oxidation run *ε*, which contained only 150 mm wafers from batch A, a homogeneous lifetime profile was observed across the oxidation furnace (see Figure 8). Except for quartz boat slot 11, the effective lifetime across each wafer ranged from 1.4 to 1.6 ms. While for quartz boat positions 6 to 10 almost similar effective lifetimes were observed, a slight lifetime decrease appears to occur in direction to the tube door. However, compared to the results of the oxidation runs *δ* and *γ*, the measured effective lifetimes are generally reduced.

**Figure 9:**
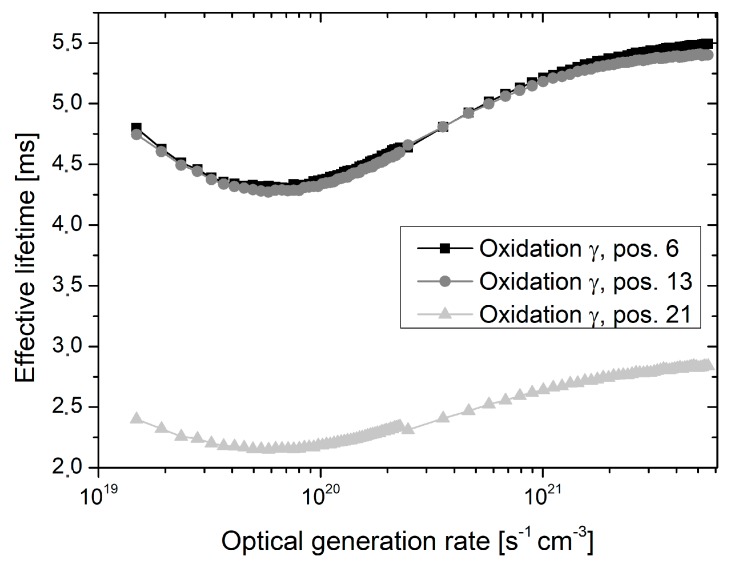
Effective lifetime as a function of the optical generation rate measured in the center of wafers, which were extracted from oxidation run *γ* and placed in quartz boat positions 6, 13, and 21, respectively. Quartz boat position 21 is located in direction of the tube door.

**Figure 10:**
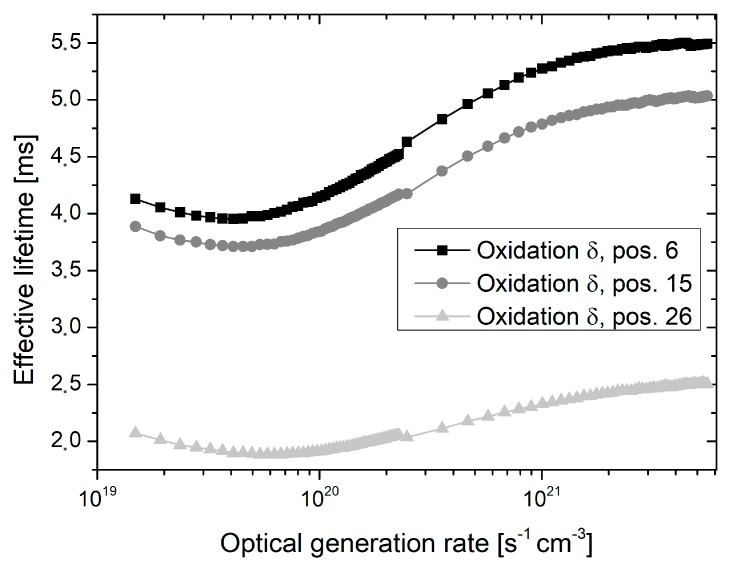
Effective lifetime as a function of the optical generation rate measured in the center of wafers, which were extracted from oxidation run *δ* and placed in quartz boat positions 6, 15, and 26, respectively. Quartz boat position 26 is located in direction of the tube door.

**Figure 11:**
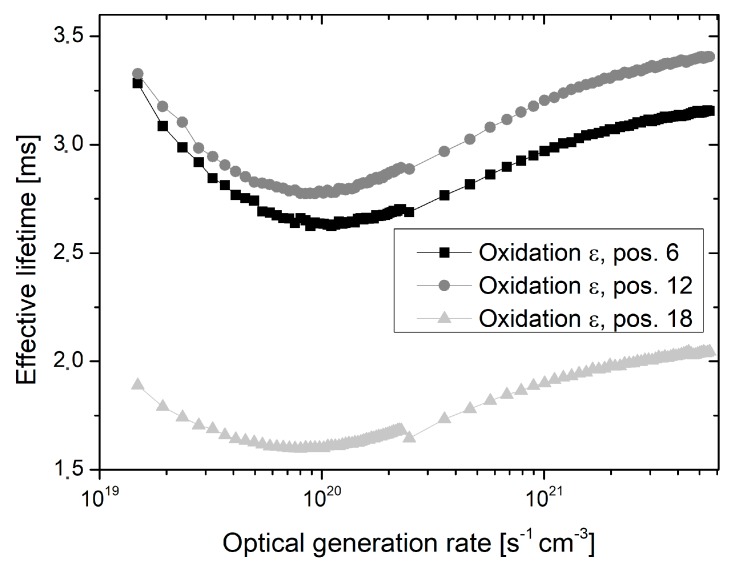
Effective lifetime as a function of the optical generation rate measured in the center of wafers, which were extracted from oxidation run *ε*, and placed in quartz boat positions 6, 12, and 18, respectively. Quartz boat position 18 is located in direction of the tube door.

The lifetime decrease towards the tube door appeared even more pronounced in the case of generation rate dependent lifetime measurements, which were performed in the center of all investigated wafers. Exemplary results for each oxidation are depicted in Figure 9, Figure 10 and Figure 11. For all optical generation rates, a significant lifetime decrease is observed at the outmost measured quartz boat slot. However, the shape of the generation rate dependent lifetime curve appears almost unchanged for all oxidations and quartz boat position. The measured effective lifetime is initially decreasing with declining optical generation rate, followed by a slight increase for low optical generation rates, which may be attributed to trapping effects [12].

**Figure 12:**
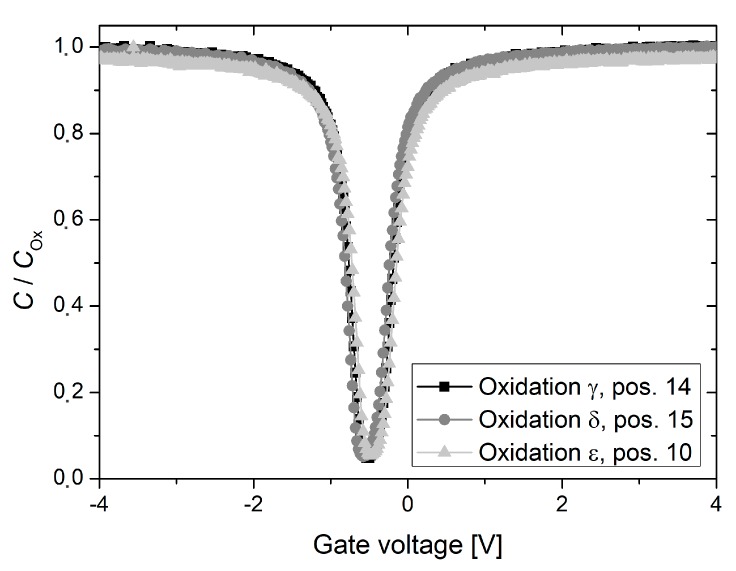
Exemplary results of quasi static capacitance-voltage measurements, which were performed on one test wafer from the oxidation runs *γ* to *ε*. Exemplary measurements were carried out at MOS capacitors (area 1 mm^2^) in the center of each wafer.

Exemplary results of the quasi static capacitance-voltage measurements, which were performed on MOS structures of one test wafer from the oxidation runs *γ* to *ε*, are illustrated in Figure 12. Almost no differences are observed between the quasi static capacitance-voltage curves, suggesting that all oxides are of high and similar quality.

## 4. Discussion

### 4.1. Oxidation Process Characterization

Fixed oxide charges as well as Si/SiO_2_ interface states are structural oxidation induced defects, which arise in every oxidation and are therefore unavoidable. However, their magnitude is significantly reduced during the post oxidation anneal under pure nitrogen atmosphere [1]. In the case of oxidation run *β*, capacitance-voltage measurements revealed high fixed oxide charge as well as enhanced Si/SiO_2_ interface densities (see Figure 5). Due to the intentionally provoked furnace leakage, ambient air entered the furnace during the entire oxidation process. In particular, post oxidation annealing was not performed under pure nitrogen atmosphere and it is assumed that oxidation induced defects were not properly annealed. In addition, ambient air and therefore oxygen was present during the cool down phase, which may have even increased the fixed oxide charge density [13]. Compared to an ideal oxidation, i.e., without intentionally provoked leakage, the measured effective lifetime was heavily affected, independent of batch and wafer diameter. Across the characterized wafers, decreased lifetimes were observed for the highest possible optical generation rate, which may be explained by higher Si/SiO_2_ interface states (see Figure 3).

For oxidation runs *α* and *β*, the effective lifetime is significantly increasing with declining optical generation rate; a behavior, which is not observed for the ideal oxidation (see Figure 4). This effect can only be explained qualitatively, since, as mentioned above, the true injection levels are not known. However, it can be assumed that the injection level decreases for declining optical generation rates. In general, the recombination at the Si/SiO_2_ interface is most effective when the ratio of the hole to electron capture cross section equals the ratio of the electron to hole carrier concentration at the interface, i.e., [14]

(7) $$\frac{\sigma_{p}}{\sigma_{n}}=\frac{n_{s}}{p_{s}}\approx \frac{1}{100}$$

For lower injection levels, band bending at the Si/SiO_2_ interface is entirely dominated by the high positive oxide charge. The Si/SiO_2_ interface is strongly accumulated by electrons and holes are effectively repelled. Since almost no holes are present at the Si/SiO_2_ interface, the condition stated in Equation (7) is violated, no effective recombination takes place, and high lifetimes are observed. For higher injection levels, the bands are flattened and the ratio of the surface excess carrier concentration becomes equal the ratio of the capture cross sections. Therefore, effective recombination can take place and lower lifetimes are observed.

In the case of the almost ideal oxidation runs *δ* and *γ*, the measured lifetime is decreasing with declining optical generation rate. Due to the moderate positive oxide charge only small band bending occurs at the Si/SiO_2_ interface. For lower injection levels, the ratio of the excess carrier concentration is assumed to be equal the ratio of the capture cross sections, which results in a high recombination activity and therefore low lifetimes. For increasing injection levels, the ratio of the surface excess carrier concentration approaches unity, thus violating the condition, which is stated in Equation (7). The result is a reduced recombination activity and hence higher lifetimes. Similar relations between injection dependent surface recombination and fixed oxide charge were predicted in the theoretical work by Otaredian [15] as well as discussed by Aberle et al. [16].

### 4.2. Furnace Profiling

For all oxidations, a distinct lifetime decrease towards the tube door of the oxidation furnace was observed (see Figure 6, Figure 7 and Figure 8). Since measurements were performed on FZ substrates from different ingots and wafers were placed randomly in the oxidation furnace, this effect is clearly attributed to an increased interface recombination, i.e., decreasing interface lifetime. In contrast, the relatively low lifetimes, which were observed across oxidation run *ε* as well as for quartz boat positions 20 and 22 of oxidation run *δ*, are assumed to originate from a reduced bulk quality. Exemplary C–V measurements suggest that no strong leakage occurred and the obtained thermal oxides are of high and almost equal quality (see Figure 12).

Despite the fact that oxidation runs *γ* to *ε* were performed by means of proper tube door sealing, small residual leakage may have still occurred and caused lower lifetimes at outer quartz boat positions. Additionally, inhomogeneous temperature profiles as well as back diffusion from the exhaust system may be an explanation. The shape of the generation rate dependent lifetime curves appears almost unchanged for all oxidations and quartz boat positions, and there is no indication for an increased fixed oxide charge concentration towards the tube door (see Figure 9, Figure 10 and Figure 11). Therefore, it is assumed that the reduced lifetimes at outer quartz boat positions are caused by an enhanced Si/SiO_2_ interface state density.

The results suggest that in this specific furnace configuration, homogenous profiles across the furnace are only achievable at the innermost quartz boat positions.

## 5. Conclusions

In the present work, contactless lifetime measurements were performed to analyze the oxidation homogeneity across a horizontal oxidation furnace. Characterization was performed by means of various FZ substrates with high bulk quality, suggesting that the measured effective lifetime is strongly dominated by interface recombination and therefore reflects the oxide quality. The presented approach was successfully used to identify strong leaks, which were intentionally provoked. In addition, it was shown that even small inhomogeneities in an otherwise ideal oxidation process can be detected with a fast and simple measurement.

The applied approach requires neither test structure nor time consuming measurements and is therefore of particular interest if high throughput is required. Since wafers are not destroyed during the characterization, the analyzed material can even be used for further device fabrication.

Characterization is not limited to the MDP technique or FZ substrates. In principle other contactless lifetime measurement methods may be used in combination with high bulk lifetime materials.

The presented technique allows a fast and accurate furnace profiling with respect to non-uniform process parameters. Besides plain equipment characterization, the presented approach is suitable to optimize the oxidation process itself regarding different parameters like temperature, gas flow, pressure, or process time.

## Figures and Tables

**Table 1:** Detailed information about the investigated material.

Batch	Dopant	Resistivity [kΩ cm]	Orientation	Thickness [µm]	Diameter [mm]
A	Phosphorus	>1.0	<100>	450	150
B	Phosphorus	>1.0	<100>	450	150
C	Phosphorus	>1.0	<100>	450	100

**Table 2:** Detailed information about the furnace loading.

Oxidation Run	Batch	Comments
*α*	B	Leaky tube door
*β*	C	Leaky tube door
*γ*	B	
*δ*	C	
*ε*	A	
